# Hypohidrotic ectodermal dysplasia and juxtaclavicular beaded lines

**DOI:** 10.1016/j.jdcr.2022.08.020

**Published:** 2022-08-22

**Authors:** Anna Dubois, William Fostier, James Sampson, Justin Durham, Neil Rajan

**Affiliations:** a Department of Dermatology, Royal Victoria Infirmary, Newcastle upon Tyne, U.K.; b Translational and Clinical Research Institute, Newcastle University, Newcastle upon Tyne, U.K.; c Department of Pathology, Royal Victoria Infirmary, Newcastle upon Tyne, U.K.; d Dental Clinical Research Unit, School of Dental Sciences, Newcastle University, Newcastle upon Tyne, U.K.

**Keywords:** ectodermal dysplasia, EDARADD, genetics, sebaceous gland, *EDA*, ectodysplasin A1 gene, *EDAR*, ectodysplasin A receptor gene, *EDARADD*, ectodysplasin A receptor-associated death domain gene, HED, hypohidrotic ectodermal dysplasia, *IKBKG*, inhibitor of nuclear factor kappa B kinase regulatory subunit gamma, JCBL, juxtaclavicular beaded lines, NF-κB, nuclear factor kappa-light-chain-enhancer of activated B-cells, *WNT10A*, wingless-type MMTV integration site family, member 10A

## Introduction

Hypohidrotic ectodermal dysplasia (HED) is characterized by abnormal development of ectodermal-derived structures and causes hypotrichosis, hypodontia, and hypohidrosis, as well as dryness of the mucosae and dysmorphic features. Here, we report a 25-year-old female patient with typical clinical features of HED and a pathogenic variant in the *EDARADD* gene, yet displaying prominent sebaceous glands around the neck, compatible with juxtaclavicular beaded lines (JCBL). JCBL has not previously been described in HED; however, isolated reports exist of patients with HED who present with enlarged facial sebaceous papules.^1^^,^^2^ Given the reduction in size and number of cutaneous ectodermal structures in this condition, the finding of prominent sebaceous glands seems unexpected and adds to the phenotypic spectrum of HED.

## Case report

**Fig 1:**
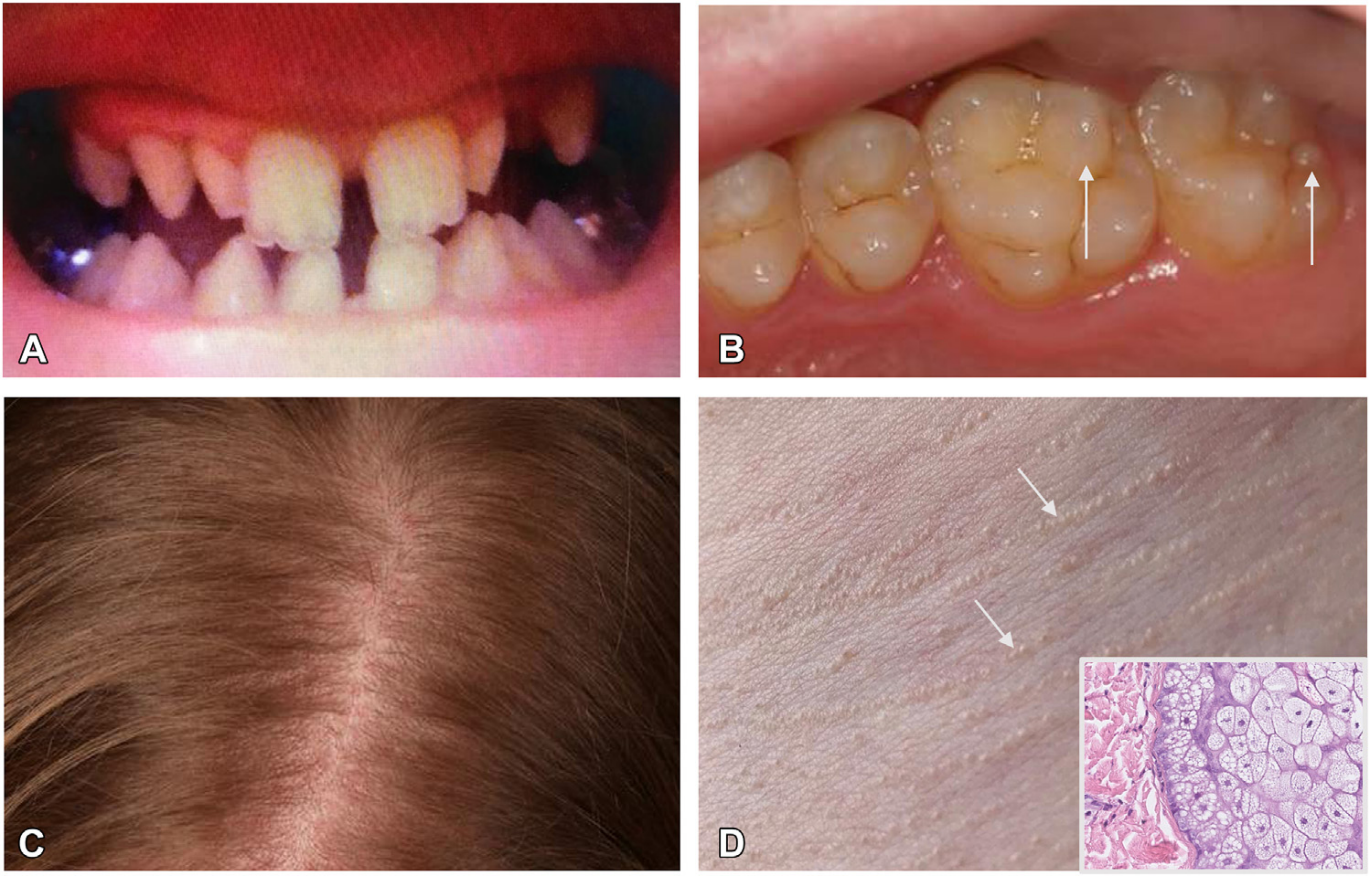
**A,** Widely-spaced peg-like and conical teeth. **B,** Five cusps on molars—*white arrows*. **C,** Thin, slightly sparse hairs on scalp. **D,** Juxtaclavicular beaded lines on the neck, with prominent sebaceous glands in skin biopsy of a papule from the neck (inset).

The proband reported a lifelong intolerance of warm environments, leading at times to dizziness and vomiting and an inability to sweat during exercise. Her deciduous teeth had grown normally, but there was delayed eruption of the permanent teeth, which were small, peg-like, and widely spaced (Fig 1, *A*- age 16); dental treatment to improve their appearance and function was undertaken later. She reported thin hair and dry skin, particularly on the hands and feet. Family history revealed symptoms of heat intolerance in her mother. On examination, although her incisors and canines had been restored, her molars had supernumerary cusps (Fig 1, *B*). Fine, relatively sparse hair was noted on the scalp (Fig 1, *C*). Her skin was dry, with a mild diffuse palmoplantar keratoderma. The nails appeared normal and there was no facial dysmorphism. Multiple concentric lines of small yellow papules were seen on the anterior chest in a necklace distribution (Fig 1, *D*). Skin biopsy of a papule demonstrated prominent sebaceous glands comprising variably sized, mature sebocytes with an intact germinative cell layer (Fig 1, *D* inset). A second biopsy from behind the ear showed sebaceous glands which largely lacked accompanying hair follicles, and there was an absence of eccrine glands in both biopsies. Elastic fibers appeared normal on elastin van Gieson stain, and von Kossa stain was negative. Genetic testing using an ectodermal dysplasia gene panel (PanelApp-UK) revealed a heterozygous pathogenic variant in the *EDARADD* gene (c.335T>G; p.Leu112Arg). Sanger sequencing of parental DNA did not show evidence of this pathogenic variant. The absence of the pathogenic variant in both parents is compatible with the variant arising *de novo* during embryogenesis or transmission from a parent with low-level mosaicism undetectable by Sanger sequencing.

## Discussion

Ninety percent of the genetic basis of HED can be explained by homozygous, hemizygous, or heterozygous pathogenic variants in 4 genes, *EDA*, *EDAR*, *EDARADD*, and *WNT10A*.^3^ These genes play important roles in the fetal development of hair, eccrine glands, and teeth.^4^ *EDA*, *EDAR*, and *EDARADD* encode interacting proteins required for NF-κB signaling, resulting in a clinically indistinguishable phenotype. *IKBKG* is an additional gene important for NF-κB signaling, and male patients with germline *IKBKG* pathogenic variants have additional clinical features including immunodeficiency, osteopetrosis, and lymphoedema.^5^

Variants in *EDARADD* account for only 1% to 2% of HED cases, and patients with heterozygous variants are recognized to have a milder phenotype than patients with homozygous variants. Previously described phenotypes in patients with homozygous variants in *EDARADD* include sparse hair, peg-shaped teeth, recurrent rhinitis with multiple respiratory infections, and dry, eczematous skin.^6^^,^^7^ Two families have been reported with autosomal dominant, heterozygous mutations in the *EDARADD* gene.^8^^,^^9^ The variant in our case (p.Leu112Arg) was previously reported in a North African family who had hypotrichosis, hypodontia, and hypohidrosis.^8^ A German family with a p.Asp123Asn pathogenic variant had additional features including absence of one or both nipples, and in addition, the proband developed mature ovarian teratomas containing hair, eccrine, and sebaceous glands at 16 years of age.^9^

Facial sebaceous gland hyperplasia has been reported in 3 hemizygous male *EDA* pathogenic variant carriers and is considered surprising, as inactivating *EDA* pathogenic variants are thought to account for the hypoplastic hair phenotype seen.^1^ The papules on our patient’s neck are consistent with previous descriptions of JCBL as yellow or skin-colored papules occurring in the relaxed skin tension lines of the anterior neck, histologically demonstrating sebaceous gland hyperplasia associated with a vellus hair. Although considered by some as a normal anatomic variant,^10^ the development of JCBL is incompletely understood and is not previously reported in conjunction with HED. The prior reports of facial sebaceous gland hyperplasia, the presence of sebaceous glands in ovarian teratomas, and this description of JCBL challenge the existing conceptual frameworks regarding the role of EDA signaling in hair and sebaceous gland homeostasis.

## Conflicts of interest

None disclosed.
